# *Symphysodontella madhusoodananii* (Pterobryaceae, Moss) a new species from the Western Ghats of India

**DOI:** 10.3897/phytokeys.18.3314

**Published:** 2012-12-06

**Authors:** C. N. Manju, K.P. Rajesh

**Affiliations:** 1 Department of Botany, the Zamorin’s Guruvayurappan College, G.A. College P.O, Kozhikode 673014, Kerala, India; 2 Malabar Botanical Garden, G.A. College P.O., Kozhikode 673014, Kerala, India

**Keywords:** *Symphysodontella madhusoodananii*, Pterobryaceae, Moss, Western Ghats, New Amarambalam, Kerala, India

## Abstract

*Symphysodontella madhusoodananii* Manju & Rajesh, **sp. nov.** an epiphytic pendant moss, with flagellate branches and long acuminate leaves with two short costa is described and illustrated from the tropical wet evergreen forests of the Western Ghats of India.

## Introduction

*Symphysodontella* Fleischer is a genus of nine species known so far from India, Sri Lanka, Myanmar, Thailand, Malaysia, Vietnam, Indonesia, Papua New Guinea, New Caledonia, Borneo and the Philippines. Magill (1980) examined this genus carefully and defined it in detail by shifting the odd members to other genera such as *Myurium* and *Pterobryopsis*. The genus is characterised by long stem, creeping on bark, usually dendroid by pinnate or bipinnate branching or long drooping, sometimes with flagelliform branches; leaves ovate-lanceolate, acuminate, concave, with short, single or double costa, leaf cells elongate-smooth. The alar is usually not differentiated, however in some species it is coloured and with porose walls. The capsule is immersed or exserted, with small, cucullate, naked, calyptra. The spores are large and papillose.

Four taxa, *viz*., *Symphysodontella borii* Dixon, *Symphysodontella pilifolia* Dixon, *Symphysodontella subulata* Broth. and *Symphysodontella tortifolia* Dixon were reported from the North-eastern India (Gangulee 1972). Of these *Symphysodontella borii* Dixon was moved to *Myurium* as *Myurium borii* (Dixon) Magill, and *Symphysodontella pilifolia* Dixon to *Pterobryopsis* as *Pterobryopsis pilifolia* (Dixon) Magill. Another related taxon, *Symphysodon involutus* (Thwaites & Mitt.) Broth. was transferred to *Symphysodontella* as *Symphysodontella involuta* (Thwaites & Mitt.) M.Fleischer (Magill 1980). Thus in India three valid species, *viz*.,*Symphysodontella subulata* Broth., *Symphysodontella tortifolia* and *Symphysodon involutus* (Thwaites & Mitt.) Broth.,were known to occur. Among these, *Symphysodontella involuta* (Thwaites & Mitt.) M.Fleischer extends upto Southern India also (Manju et al. 2008, Daniels 2010).

During our recent exploration in the *Shola* forests (Southern montane wet temperate forests) of New Amarambalam Reserve Forest in the Malappuram District of Kerala, we collected an interesting species of *Symphysodontella*, which showed distinguishing characteristics from the other known species. We here describe this entity as a new species and provide a table of comparison with the other known species to India as well as a key.

## Taxonomic treatment

### Symphysodontella madhusoodananii

Manju & Rajesh sp. nov.

http://species-id.net/wiki/Symphysodontella_madhusoodananii

#### Diagnosis.

*Symphysodontella madhusoodananii* is characterised by very short, double costate, oblong-lanceolate, long acuminate leaves. It shows some similarities to *Symphysodontella tortifolia* Dixon in its long acuminate, complanate plicate leaves and some leaves being tortuose at tip. However, it differs in its oblong-lanceolate leaves, two distinct short costa in both main and secondary branch leaves and the leaves on main shoot and secondary branches being similar. It also differs having long drooping primary branches, short secondary branches and presence of copious flagelliform branches. *Symphysodontella madhusoodananii* also shows some similarity with *Symphysodontella subulata* Broth. in its two short-costate, ovate-lanceolate leaves, elongate-linear porose cells and yellowish brown stem attachment cells. However, it differs in cells being highly porose in its lateral wall and in the middle layer, the pores being rounded.

#### Type.

**INDIA.** Kerala, Malappuram district, Nilambur, New Amarambalam Reserve Forest (bordering Mukurti National Park of Tamil Nadu), epiphytic on trees of shola forest, 1200 m alt., *K.P.Rajesh* 106933 (Holotype: CALI! Isotypes: BM!, CAL!, CALI!, MBGS!, ZGC!).

#### Description.

**Figure 1.**
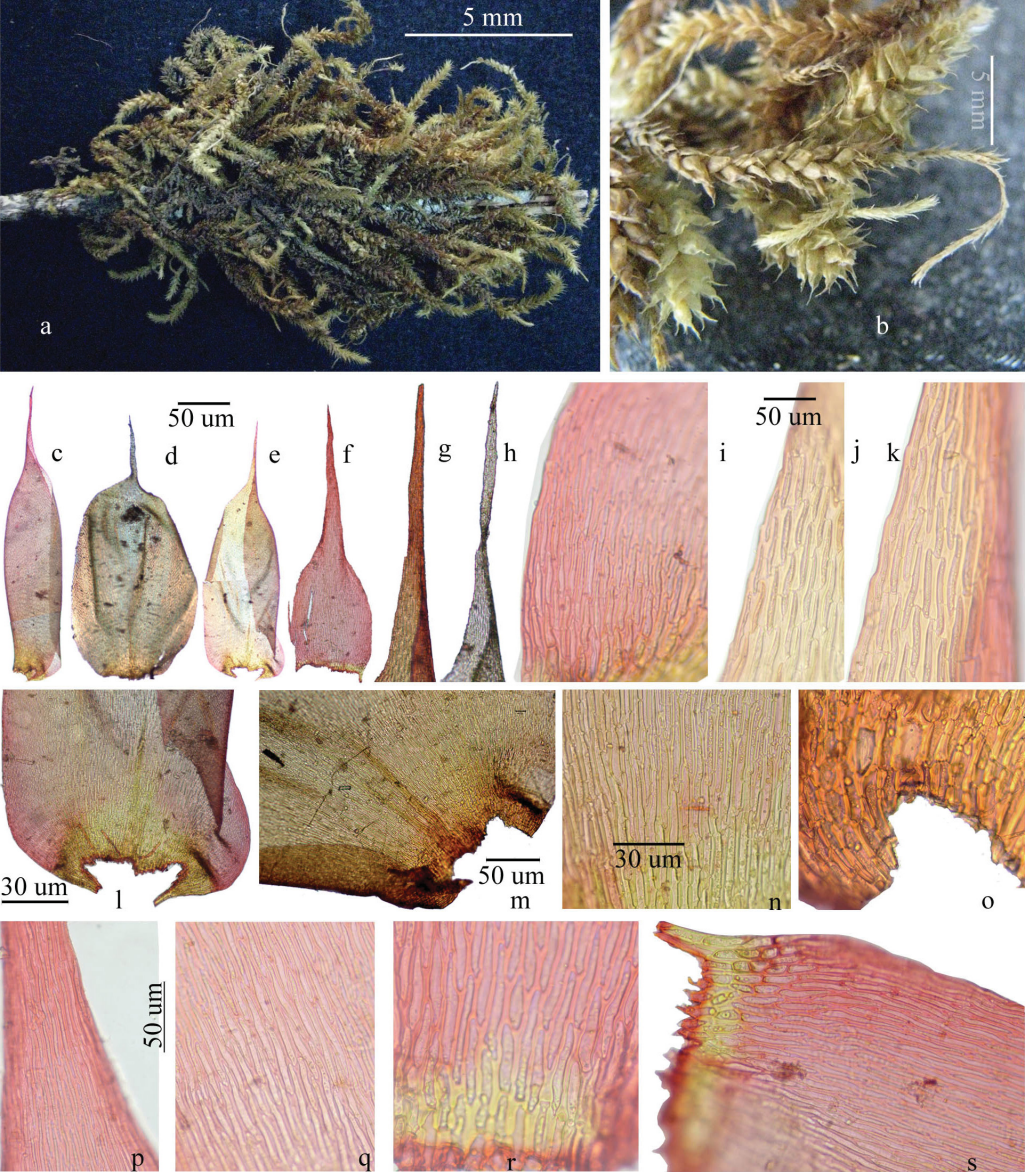
*Symphysodontella madhusoodananii*, **a–b** habit **c–e** leaf at main stem **f** leaf at flagella **g–h** leaf tip **i** basal marginal cells **j–k** leaf margin at tip **l–m** leaf insertion cells **n** leaf middle cells **o** basal cells enlarged **p** leaf tip cells **q** leaf middle cells **r–s** leaf basal cells.

Main stem long, creeping on bark, 3–5 cm long, scale leaves present, branches yellowish green to brownish, primary branch 5–7 cm long, secondary branches up to 2 cm long, mostly with flagelliform branches, flagella 2.5 cm long, erect to drooping, leaves oblong-lanceolate, lax erect-spreading, 2.5 × 0.9 mm, leaves on main shoot lax, ovate, long acuminate, acumen 0.9 mm long, two very short distinct costa at base, leaves on main shoot and secondary branches dense, patent to squarrose, complanate, plicate, tip tortuose in some leaves, long apiculate, margin denticulate at tip, recurved below, cells at acumen elongate linear, up to 50 µm long, thick walled above, middle cells 60–70 µm × 20–25 µm, less thick but with porose walls below, 35–45 µm × 28–35 µm, costa and cells at stem attachment yellowish brown, alar cells not prominently differentiated in size but with deep brown, rectangular porose cells, 40–45 × 25–35 µm, porose in some cells, leaf insertion to the stem is U shaped; cells at flagella almost same size and shape; sporophyte not seen (Figure 1 a–s).

#### Distribution.

It is distributed in the shola forests (Southern montane wet temperate forests) of New Amarambalam of Kerala and Mukuruty National Park of Tamil Nadu in the Western Ghats of India.

#### Ecology.

It was found growing as epiphytic on branches of trees of shola forest.

#### Etymology.

It is named in honour of Professor (Dr.) P.V. Madhusoodanan, for his meritorious contribution towards botany, especially on lower groups of plants of Southern India. He retired recently from the Department of Botany of University of Calicut, and now continues his research as an Emeritus Scientist in the Malabar Botanical Garden (MBG), Kozhikode. It is he who inspired the present authors to undertake studies on the bryophytes and pteridophytes.

## Discussion

*Symphysodontella madhusoodananii* is similar to *Symphysodontella tortifolia* in its long acuminate, complanate plicate leaves and some leaves being tortuose at tip. It differs from *Symphysodontella tortifolia* in its oblong-lanceolate leaves, two distinct, short costa in both main and secondary branch leaves and the leaves on main shoot and secondary branches being similar. In *Symphysodontella tortifolia* the costa in the secondary branch leaf is single, reaching a little more than half the length. The secondary branches are dendroid by bi-pinnate branching with branching in different planes in *Symphysodontella tortifolia*, and lacks flagelliform branches. However, in *Symphysodontella madhusoodananii* the primary branches are long drooping, secondary branches short and flagelliform branches are frequent.

*Symphysodontella madhusoodananii* also shows some similarity with *Symphysodontella subulata* Broth. in its two short costa and ovate-lanceolate leaves. However, in *Symphysodontella madhusoodananii* the cells are highly porose in its lateral wall and in the middle layer the pores are rounded. It is also having hanging secondary branches and copiously flagellate. The costa and cells at stem attachment is yellowish brown, alar cells not prominently differentiated but with deep brown, rectangular porose cells and the leaf insertion is U-shaped. Gangulee (1972) commented that the Indian population of *Symphysodontella subulata* Broth. scarcely shows any flagellate branch. However, such branches are reported from the Philippine populations, but with having single costa. The present species is characterised by the leaves being oblong-lanceolate, and with very short and double costa (Table 1). A key to the Indian species of *Symphysodontella* is also given for easy identification.

**Table 1.** Comparison of the morphological features of *Symphysodontella* species in India.

Characters	*Symphysodontella madhusoodananii*	*Symphysodontella subulata*	*Symphysodontella tortifolia*	*Symphysodontella involuta*
**Branching**				
Primary branches	long drooping	elongate	short	short
Secondary branches	short	hanging	dendroid	long, drooping
Flagelliform branches	frequent	frequent	absent	absent
**Leaves**				
Shape	Oblong lanceolate, long acuminate, complanate plicate	Ovate-lanceolate, acumen short	Ovate, long acuminate, complanate plicate	Lanceolate, not plicate
Apices	some leaves tortuose at tip	leaf tip not tortuose	some leaves tortuose at tip	Tip involutus; not tortuose at tip
Costa	Two short, distinct in both main and secondary branches	Very short two costa, not distinct	Single, reaching a little more than half the length in the secondary branch leaves	Single, reaching the middle of leaf, distinct in main and secondary branches
**Cells**				
Shape	elongate linear	elongate linear	elongate	elongate
wall	highly porose in its lateral wall and in the middle layer, pores rounded	thick walled, porose, rounded	slightly thick walled above, weekly porose above and less thick but porose walls below	Thin walled above and middle, thick porose
alar cells	not prominently differentiated in size but with brown cells, rectangular porose cells	differentiated with deep brown, rectangular porose cells	Tinted, less thick cells, rectangular	thick porose walls, tinted, rectangular

**Conservation status.** The species was found growing on the trees of shola forests (Southern montane wet temperate forests) of New Amarambalam of Kerala, and the adjoining areas of Mukuruty National Park of Tamil Nadu. Located in the Nilgiri Biosphere Reserve, the New Amarambalam Reserved Forests with an area of more than 260 km^2^, is one of the most notable areas in the Western Ghats of Northern Kerala for its unique assemblage of floristic elements. The rare blending of many interesting floral elements makes this area as one among the most potential area to be considered for long term conservation. The unique geographic location and the rugged terrain with folds of hills and deep valleys, as a natural westward extension of the Nilgiri hills make this landscape dotted with all major vegetation types of Southern India in a short span itself. The proposed plan for designating the New Amarambalam forests of Kerala as a Wildlife Sanctuary has not been materialised yet. The area with its unique geographical location, which changes along a sharp altitudinal gradient and thus offering a good array of natural habitats, forming the rare blend of species assemblages, is no doubt, an ideal landscape to be conserved. This also ensures the long term protection of many species and their populations, in a unique landscape with the continuum of the Nilgiri hills to the Silent Valley and adjacent areas. At present the species is known only from a small area, and with the majority lies in the non-protected part. Its conservation status could be improved by bringing these areas of Kerala state under protection.

**Specimens examined**: INDIA. Kerala, Malappuram district, Nilambur, New Amarambalam forest (bordering the Mukurti National Park of Tamil Nadu), epiphytic on trees of shola forest, 1200 m alt., *K.P. Rajesh* 106933 (BM!, CAL!, CALI!, MBGS!, ZGC!); New Amarambalam Reserve Forest, 1200 m alt., *K.P. Rajesh* 111862 (CALI!, MBGS!, ZGC!); INDIA. Tamil Nadu, Pandiyar Estate (near Mukurthi National Park), 1300 m alt., on branches of trees of shola forest, *K.P. Rajesh* 111852 (CALI!, MBGS!, ZGC!), Mukurthi National Park, 1400 malt., on branches of trees of shola forest, *K.P. Rajesh* 109001 (CALI!, MBGS!, ZGC!).

### Key to the species of *Symphysodontella* in India

**Table:** 

1a	Leaves complanate plicate, acumen long, 1 mm	2
1b	Leaves not plicate, acumen very short, 02-0.6 mm	3
2a	Costa in the primary and secondary branch leaves single, reaching a little more than half the length; flagelliform branches absent	*Symphysodontella tortifolia*
2b	Costa in the primary and secondary branch leaves double, short, restricted to alar region; flagelliform branches frequent	*Symphysodontella madhusoodananii*
3a	Plants smaller, 4 cm long; leaf tip narrow subulate	*Symphysodontella subulata*
3b	Plants robust, 8–10 cm long; leaf tip involutus	*Symphysodontella involuta*

## Supplementary Material

XML Treatment for Symphysodontella madhusoodananii
